# Shorter Food Chain Length in Ancient Lakes: Evidence from a Global Synthesis

**DOI:** 10.1371/journal.pone.0037856

**Published:** 2012-06-06

**Authors:** Hideyuki Doi, M. Jake Vander Zanden, Helmut Hillebrand

**Affiliations:** 1 Institute for Chemistry and Biology of the Marine Environment, Carl-von-Ossietzky University Oldenburg, Schleusenstrasse 1, Wilhelmshaven, Germany; 2 Institute for Sustainable Sciences and Development, Hiroshima University 1-3-1 Kagamiyama, Higashi-Hiroshima, Japan; 3 Center for Limnology, University of Wisconsin, Madison, Wisconsin, United States of America; Dalhousie University, Canada

## Abstract

Food webs may be affected by evolutionary processes, and effective evolutionary time ultimately affects the probability of species evolving to fill the niche space. Thus, ecosystem history may set important evolutionary constraints on community composition and food web structure. Food chain length (FCL) has long been recognized as a fundamental ecosystem attribute. We examined historical effects on FCL in large lakes spanning >6 orders of magnitude in age. We found that food chains in the world’s ancient lakes (n = 8) were significantly shorter than in recently formed lakes (n = 10) and reservoirs (n = 3), despite the fact that ancient lakes harbored much higher species richness, including many endemic species. One potential factor leading to shorter FCL in ancient lakes is an increasing diversity of trophic omnivores and herbivores. Speciation could simply broaden the number of species within a trophic group, particularly at lower trophic levels and could also lead to a greater degree of trophic omnivory. Our results highlight a counter-intuitive and poorly-understood role of evolutionary history in shaping key food web properties such as FCL.

## Introduction

Food chain length (FCL), which is a measure of the number of trophic levels in an ecosystem between primary producers and the top predator, has been recognized as a fundamental ecosystem attribute. FCL influences community structure, species diversity, and population stability by altering the organization of trophic interactions [1]–[2]. Defining the factors that determine FCL has been an important research focus for ecologists. Numerous widely cited hypotheses have been proposed. The productivity hypothesis predicts that energy availability limits FCL [1]–[2], due to limited efficiency in energy transfer up the food chain, such that the available energy diminishes at higher trophic levels. The ecosystem size hypothesis predicts that FCL increases with increasing ecosystem size, such as lake volume and island area [3]. The productive-space hypothesis [4] is a combination of the productivity and the ecosystem size hypotheses. This argues that total ecosystem production (per-unit-area productivity × ecosystem size) reflects the productive capacity of the ecosystem to support higher trophic levels. The dynamic stability hypothesis argues that long food chains tend to be dynamically unstable in the face of disturbance, such that food webs should be shorter in more highly-disturbed systems [5]–[6].

Ecosystem history may set important evolutionary constraints on ecological processes and properties of ecosystems [7]–[8]. Food webs are affected by evolutionary processes such as speciation, and effective evolutionary time ultimately affects the probability of species evolving to fill the available niche space [9]. In addition, a long evolutionary history may provide more opportunities for food web disturbances. Previous studies have shown that FCL varies greatly among systems [3], [10]–[13]. However, the role of ecosystem age and the historical signal on FCL has not been examined.

How might FCL vary as a function of ecosystem age? First, older ecosystems might be expected to have longer food chains, as speciation and colonization should increase overall biodiversity, and thus the increase the potential to assemble longer food chains [11]. On the other hand, speciation could lead to an increase in herbivores and other low trophic position species. This might be expected since shorter food chains are expected to be more dynamically stable [14]. There are numerous examples of evolutionary adaptations towards lower trophic position, for example, the filter feeding apparatus of baleen whales, which evolved from the teeth of toothed whales [15]. If speciation simply increases the number of herbivorous species or increases the frequency of intraguild predation [16], older ecosystems could end up with shorter, not longer, food chains.

**Table 1:** Dataset used for the present study comprising lake name.

Name	Origin	Surface area (km^2^)	Volume (km^3^)	Mean depth (m)	Max. depth (m)	Latitude	Age of lake (year)	Endemicspecies (N)	FCL	δ^15^N_ tp-predator_ (‰)	δ^15^N_baseline_ (‰)	Name of toppredator	Datasource to calculate FCL
Albert	Tectonic	5,600	280	25	58	1.4	4,000,000	9	3.88	11.6	5.2	*Lates nilotica*	[26]
Baikal	Tectonic	31,500	23,600	730	1637	53.6	27,500,000	982	4.18	13.9	6.5	*Comephorus baicalensis*	[27]–[28]
Biwa	Tectonic	670	27.5	43	104	35.2	1,000,000	54	3.92	17.4	10.9	*Silurus biwaensis*	[29]
Kyoga	Tectonic	1,300	3.2	6	8	6.0	400,000–14,600	100	3.79	9.1	3.0	*Rastrineobola argentea*	[30]
Malawi	Tectonic	29,500	7,775	264	706	S 12.0	10,000,000	620	3.68	8.4	2.7	*Rhamphochromis ferox*	[31]
Tahoe	Tectonic	495	156	305	501	39.0	2,000,000	7	3.55	-	-	*Salmo trutta*	[32]
Tanganyika	Tectonic	32,600	19,000	570	1470	6.0	3,000,000	632	3.59	13.2	7.8	*Polypterus congicus*	[33]
Victoria	Tectonic	68,870	2,760	40	80	S 1.3	400,000–14,600	700	3.68	12.7	7.0	*Bagrus docmac*	[34]
Champlain	Glacier	1,127	25.8	20	122	19.5	10,000	0	4.85	18.9	9.2	*Salvelinus namaycush*	[3]
Chany	Glacier	2,500	4.3	2	6	54.5	20,000	0	4.18	13.0	5.6	*Perca fluriatilus*	[35]–[36]
Erie	Glacier	25,700	484	19	64	41.7	13,000	0	4.39	17.3	9.2	*Salvelinus namaycush*	[3]
Gender	Glacier	113	11.9	-	288	48.6	11,800	0	4.51	12.9	4.3	*Salvelinus alpinus*	[37]
Great Slave	Glacier	26,915	2,089	41	614	61.5	20,000	0	5.28	13.5	2.3	*Salvelinus namaycush*	[38]
Michigan	Glacier	57,800	4,920	85	282	44.0	11,800	0	4.10	16.4	9.2	*Lota lota*	[39]
Ontario	Glacier	18,960	1,640	86	241	43.5	11,800	0	5.02	18.4	8.1	*Salvelinus namaycush*	[3]
Pend Oreille	Glacier	350	53.9	-	366	48.1	9,000	0	4.49	15.6	7.2	*Salvelinus confluentus*	[40]
Superior	Glacier	82,100	12,100	147	406	47.5	9,000	0	4.15	10.3	3.0	*Salvelinus namaycush*	[41]
Winnipeg	Glacier	24,500	371	12	36	53.0	10,000	0	4.20	18.0	10.6	*Stizostedion canadense*	[42]
Nasser	Reservoir	6,000	157	25	130	22.5	37	0*	4.38	12.1	4.0	*Clarias* sp.	[43], unpublished data
Roosevelt	Reservoir	307	11.7	-	114	48.2	66	0*	4.04	12.2	5.3	*Lota lota*	[44]
Shasta	Reservoir	741	5.8	-	158	40.8	63	0*	4.19	11.9	4.4	*Oncorhynchus tshawytscha*	[45]

Age of lakes indicate the averages of age with refer to recent literatures. Endemic species indicate observed and predicted endemic species number with refer to recent literatures. δ^15^N_top-predator_ and δ^15^N_baseline_ indicates mean values.

* Our assumption, because of short history of the reservoir.

Here we investigate patterns of FCL using a global lake dataset. Lakes in our data set vary in size by >5 orders of magnitude, and in age from a few to 10^7^ years, depending on whether they are tectonic, glacial or impounded lakes. Ancient lakes (typically >1 million year) have a long evolutionary history and consequently higher species diversity, often with a large number of endemic species. In the present study, we used stable nitrogen isotope-based estimates of FCL from reservoirs, glacial, and ancient lakes. Our data set includes 68% of all freshwater ancient lakes >1 million years in age. Since some empirical studies suggest that ecosystem size is a driver of FCL [3], we compared FCL in ancient lakes to more recent lakes within a similar range of lake volumes.

**Figure 1:**
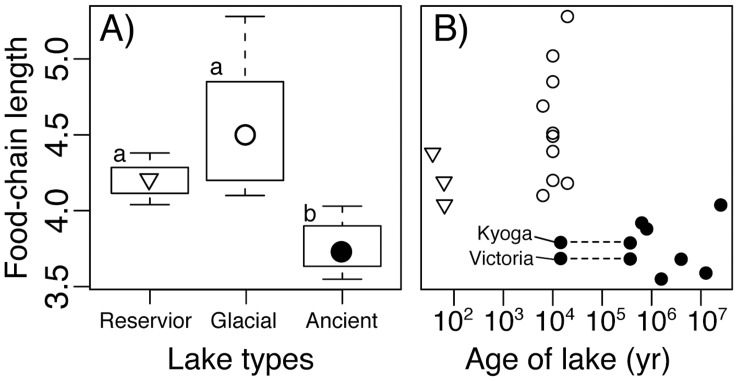
Food-chain length in lakes of different origins and age. A) Boxplot for food-chain length in lakes of different origins. Symbols represent the median FCL, boxes  =  inter-quartiles, and bars  =  maximum and minimum values. Different letters indicate significant differences by multiple comparison using ANOVA (p<0.001). B) Correlation between age of lake and food-chain length. Different symbols are for reservoir, glacial and ancient lakes (see A). Lake Victoria and Lake Kyoga are originally from 400,000 years ago, but are thought to have dried up 14,600 years ago.

## Materials and Methods

### Data Searching

We searched for stable isotope datasets from published papers using ISI Web of Science and Google Scholar. We used “isotope”, “lake” and lake and reservoir names such as "Baikal" as keywords. Lakes and reservoirs were selected using the published literature [17] and the World Lake Database, which includes the large lakes of the world (http://www.ilec.or.jp/database/database.html, and LakeNet; http://www.worldlakes.org/searchlakes.asp). We selected papers that report nitrogen stable isotope values of top predator fishes (seal in Lake Baikal) and invertebrate primary consumers such as zooplankton, mussels, and aquatic benthic invertebrates. We found suitable nitrogen isotope data for 21 large lakes and reservoirs from 23 published and unpublished papers. A list of lakes and reservoirs included in this analysis, and the relevant lake attribute data and literature references are in Table 1. A strong relationship between FCL and ecosystem size has been found among temperate lakes [3]. To avoid confounding effects of ecosystem size, we only included recent lakes and reservoirs larger than 3.2 km^3^, which was the volume of the smallest ancient lake included in the present study (Table 1).

### Estimation of FCL

FCL for a given lake is defined as the trophic position of the fish species with the highest mean δ^15^N value. We assumed a trophic fractionation of 3.4‰ to calculate FCL as in previous studies [3], [10]. For each food web, we estimated FCL using stable nitrogen isotope as follows [3], [10]:
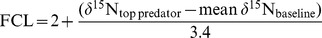
where the mean δ^15^N of invertebrate primary consumers (zooplankton, mollusks, and other benthic invertebrates) were used as the isotopic baseline. As in [11], we used stable isotope information from all primary consumers from both pelagic and benthic habitats for the δ^15^N_baseline_. The observed variation in δ^15^N_baseline_ did not significantly affect the estimation of FCL in lakes [11].

**Figure 2:**
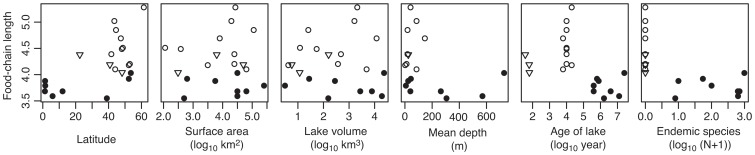
Correlations between food-chain length, latitude, log_10_ surface area (km^2^), log_10_ volume (km^3^), mean depth (m), log_10_ age of lake (year, the points of Lake Kyoga and Victoria were plotted as 400,000 year), and log_10_ (endemic species number +1). Symbols mean the origin of lakes as Fig.1A.

### Statistical Analysis

We tested for differences among the three lake types using analysis of variance (ANOVA, α = 0.05). We performed the Bonferroni adjustment for multiple comparisons as a post-hoc test in ANOVA among the three lake types (α = 0.05). To compare factors that are correlated with FCL, we used Pearson's correlation coefficients (α = 0.05). We included Lake Kyoga and Victoria as ancient lakes, because the lake basins have a long history (>400,000 years) and many endemic species, although they are suspected to have dried up 14,600 years ago [18]. All statistical tests and graphics were performed by R ver. 2.13.0 software [19].

## Results

We found that FCL in ancient lakes was significantly shorter than glacial lakes and reservoirs (ANOVA, p<0.001, multiple comparison, p<0.001, Fig. 1A). The shortest food chains were consistently in the oldest lakes (Fig. 1B). FCL of reservoirs tended to be shorter than that of glacial lakes, although the difference was not significant (Fig. 1A). FCL was not correlated with lake area, lake volume, or mean depth (Pearson's correlation coefficient, |r| <0.35, p>0.16, Fig. 2), but increased with latitude, as many ancient lakes are tropical (r = 0.60, p<0.05, Fig. 2). There was a strong positive relationship between lake age and number of endemic species (r = 0.81, p<0.001).

Lake Victoria and Lake Kyoga are located in ancient lake basins, but are suspected to have dried up 14,600 years ago (Table 1), though notably they have approximately 700 and 100 endemic Cichlid fish species, respectively. Five of the eight ancient lakes contain an endemic top predator (Table 1). Despite the fact that the long evolutionary history has led to the evolution of new and endemic top predators in many of these lakes, FCL in ancient lakes is still short relative to lakes of more modern origin.

## Discussion

We found that FCL in ancient lakes was significantly shorter than glacial lakes and reserviors, even though these ancient lakes tend to have much higher fish species richness and endemism than their more recent counterparts. Though we cannot resolve the causal mechanism as to why ancient lakes have shorter food chains, there are several potential ways in which this could occur. For example, speciation could simply broaden the number of species within a trophic group, particularly at lower trophic levels. Additional species richness does not necessarily lead to longer food chains. Furthermore, speciation could lead to a greater degree of trophic omnivory, such that FCL would naturally decline with increasing species richness. Longer time for speciation in ancient lakes may increase the number of trophic omnivores and trophic specialists relative to recent lakes. A theoretical study by Loeuille and Loreau [9] predicted that long evolutionary history would tend to decrease FCL when competition intensity is low. A high diversity of ecological specialists might decrease competition intensity, and consequently FCL of the system [9]. The other hypothesis to explain shorter FCL in ancient lakes is that speciation simply leads to more herbivores in the ecosystem [20]. In fact, African ancient lakes tend to have many herbivorous cichlid species. This is the case even in Lake Victoria and Kyoga, which are thought to have dried up 14,600 years ago [20]–[21]. Similarly, Lake Baikal has many species of zooplankton-feeding pelagic sculpins (e.g., *Cottocomephorus inermis* and *grewingki*) which evolved from benthic invertebrate-feeding sculpins [22]. To mechanistically test the above hypotheses, a potential approach could involve rapid diversification in experimental systems, e.g. involving bacteria and microbial food webs.

Cohen and Newman’s cascade model [23] predicts that increasing species richness would tend to increase FCL by increasing the total number of both nodes and links, and thus the mean path length leading to the top predator. In fact, most of the ancient lakes have high species richness and many endemic species, including endemic top predators. However, higher species richness does not correspond with elevated FCL. The dynamic constraints hypothesis predicts that more frequent or more intense disturbance would tend to shorten FCL based on simple theoretical models suggest that longer chains are less resilient, and thus unlikely to persist in the face of disturbance [5]. If ancient lakes have been more frequently disturbed relative to recent lakes, this could result in shorter food chains. Though ancient lakes have been around longer, the ecosystems tend to be large, deep and located in the tropics. Such factors may mediate or buffer the effects of disturbance on these lakes. A number of food web studies in both the laboratory and field did not find an effect of disturbance on FCL [24]–[25], possibly suggesting a limited role for disturbance history in affecting FCL.

Though it is perhaps impossible to measure the FCL of a single lake over a long time period, our comparative results suggest that lake food chains lengthen over time periods of hundreds to thousands of years as a stable biological community assembles. In contrast, over time periods of thousands to millions of years, food chains actually become shorter as species diversity rises and endemic species emerge. Because our findings are observational and comparative in nature, we ultimately cannot infer mechanisms that underlie this pattern. Nevertheless, the relationship between FCL and ecosystem age is striking. Our study is a first step towards understanding the role of historical and evolutionary factors in determining fundamental food web properties such as FCL.
